# 4,5-Dimethyl-1,2-diphenyl-1*H*-imidazole monohydrate

**DOI:** 10.1107/S1600536810030072

**Published:** 2010-08-04

**Authors:** P. Gayathri, A. Thiruvalluvar, K. Saravanan, J. Jayabharathi, R. J. Butcher

**Affiliations:** a PG Research Department of Physics, Rajah Serfoji Government College (Autonomous), Thanjavur 613 005, Tamilnadu, India; b Department of Chemistry, Annamalai University, Annamalai Nagar 608 002, Tamilnadu, India; c Department of Chemistry, Howard University, 525 College Street NW, Washington, DC 20059, USA

## Abstract

In the title compound, C_17_H_16_N_2_·H_2_O, the imidazole ring is essentially planar [maximum deviation = 0.0037 (7) Å]. The imidazole ring makes dihedral angles of 80.74 (7) and 41.62 (7)° with the phenyl rings attached to the N and C atoms, respectively. The dihedral angle between the two phenyl rings is 75.83 (8)°. Inter­molecular O—H⋯N and O—H⋯O hydrogen bonds are found in the crystal structure.

## Related literature

For related crystal structures and applications of imidazole derivatives, see: Gayathri *et al.* (2010*a* ▶,*b* ▶).
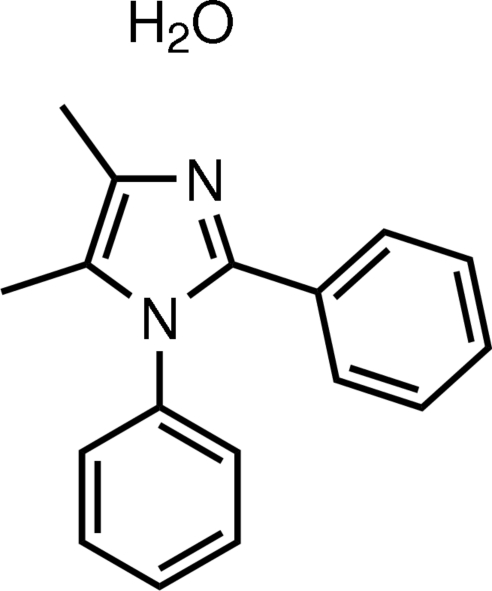


## Experimental

### 

#### Crystal data

C_17_H_16_N_2_·H_2_O
*M* *_r_* = 266.33
Tetragonal, 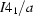

*a* = 25.5498 (2) Å
*c* = 9.3792 (1) Å
*V* = 6122.67 (9) Å^3^
*Z* = 16
Cu *K*α radiation
μ = 0.57 mm^−1^
*T* = 295 K
0.53 × 0.42 × 0.18 mm

#### Data collection

Oxford Diffraction Xcalibur Ruby Gemini diffractometer
Absorption correction: multi-scan (*CrysAlis PRO*; Oxford Diffraction, 2010 ▶) *T*_min_ = 0.805, *T*_max_ = 1.000
8078 measured reflections
3109 independent reflections
2610 reflections with *I* > 2σ(*I*)
*R*_int_ = 0.019

#### Refinement

*R*[*F* ^2^ > 2σ(*F* ^2^)] = 0.043
*wR*(*F* ^2^) = 0.134
*S* = 1.08
3109 reflections
189 parameters
H atoms treated by a mixture of independent and constrained refinement
Δρ_max_ = 0.16 e Å^−3^
Δρ_min_ = −0.21 e Å^−3^

### 

Data collection: *CrysAlis PRO* (Oxford Diffraction, 2010 ▶); cell refinement: *CrysAlis PRO*; data reduction: *CrysAlis PRO*; program(s) used to solve structure: *SIR2004* (Burla *et al.*, 2005 ▶); program(s) used to refine structure: *SHELXL97* (Sheldrick, 2008 ▶); molecular graphics: *ORTEP-3* (Farrugia, 1997 ▶); software used to prepare material for publication: *PLATON* (Spek, 2009 ▶).

## Supplementary Material

Crystal structure: contains datablocks global, I. DOI: 10.1107/S1600536810030072/tk2693sup1.cif

Structure factors: contains datablocks I. DOI: 10.1107/S1600536810030072/tk2693Isup2.hkl

Additional supplementary materials:  crystallographic information; 3D view; checkCIF report

## Figures and Tables

**Table 1:** Hydrogen-bond geometry (Å, °)

*D*—H⋯*A*	*D*—H	H⋯*A*	*D*⋯*A*	*D*—H⋯*A*
O1*W*—H1*W*⋯N3^i^	0.914 (18)	2.010 (18)	2.9111 (14)	168.6 (16)
O1*W*—H2*W*⋯O1*W*^i^	0.86 (2)	2.03 (2)	2.8957 (15)	175 (2)

Symmetry code: (i) 
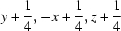
.

**Fig. 1.**
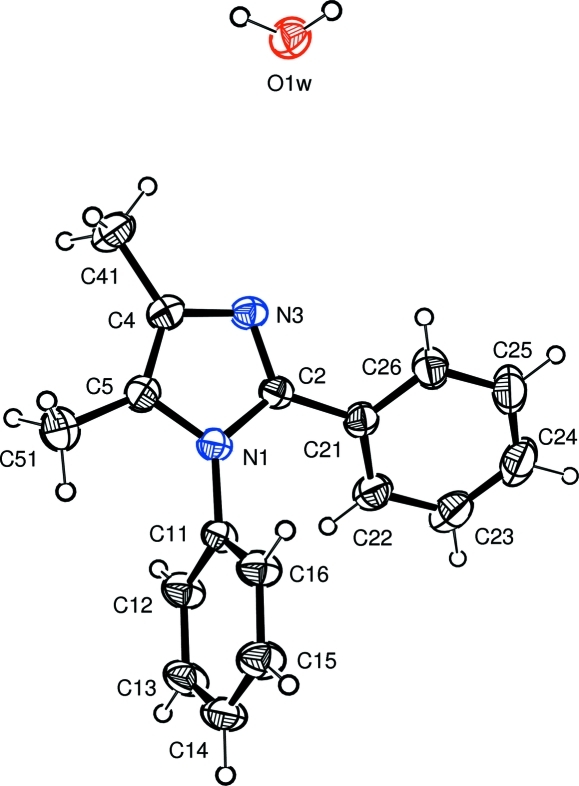
The molecular structure of the title compound, showing the atom-numbering scheme and displacement ellipsoids drawn at the 30% probability level. H atoms are shown as small spheres of arbitrary radius.

**Fig. 2.**
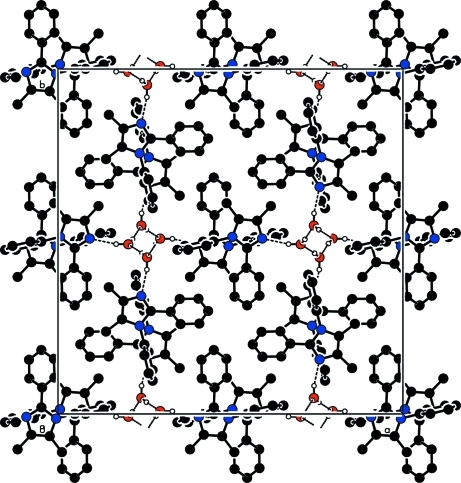
The packing of the title compound, viewed down the c axis. Dashed lines indicate hydrogen bonds. H atoms not involved in hydrogen bonding have been omitted.

## supplementary crystallographic information

### Comment

As part of our research (Gayathri *et al.*, 2010*a*,*b*), we have synthesized the title compound (I) and report its crystal structure here.

In the title compound (Fig. 1), the imidazole ring is essentially planar [maximum deviation of 0.0037 (7) Å for C5]. The imidazole ring makes dihedral angles of 80.74 (7) and 41.62 (7) ° with the phenyl ring (C11—C16) attached to N1 and the phenyl ring (C21—C26) attached to C2, respectively. The dihedral angle between the two phenyl rings is 75.83 (8) °. Intermolecular O1W—H1W···N3 and O1W—H2W···O1W hydrogen bonds are found in the crystal structure (Table 1, Fig. 2).

### Experimental

To pure butane-2,3-dione (1.48 g, 15 mmol), aniline (1.36 g, 15 mmol) and ammonium acetate (1.15 g, 15 mmol) in in ethanol (10 ml) was added benzaldehyde (1.5 g, 15 mmol) over about 1 h with the temperature maintained at 333 K. The reaction mixture was refluxed for 7 days and extracted with dichloromethane. The solid separated was purified by column chromatography using hexane:ethyl acetate as the eluent. Yield: 1.79 g (48%).

### Refinement

H1W and H2W attached to O1W were located in a difference Fourier map and refined freely. The remaining H atoms were positioned geometrically and allowed to ride on their parent atoms, with C—H = 0.93 – 0.96 Å; *U*_iso_(H) = k*U*_eq_(C), where k = 1.5 for methyl and 1.2 for all other H atoms. The methyl groups were found to be disordered over two positions. They were each refined as an idealized disordered methyl group.

### Crystal data

**Table:**

C_17_H_16_N_2_·H_2_O	*D*_x_ = 1.156 Mg m^−^^3^
*M**_r_* = 266.33	Melting point: 375 K
Tetragonal, *I*4_1_/*a*	Cu *K*α radiation, λ = 1.54184 Å
Hall symbol: -I 4ad	Cell parameters from 4027 reflections
*a* = 25.5498 (2) Å	θ = 4.9–77.4°
*c* = 9.3792 (1) Å	µ = 0.57 mm^−^^1^
*V* = 6122.67 (9) Å^3^	*T* = 295 K
*Z* = 16	Irregular plate, colourless
*F*(000) = 2272	0.53 × 0.42 × 0.18 mm

### Data collection

**Table:**

Oxford Diffraction Xcalibur Ruby Gemini diffractometer	3109 independent reflections
Radiation source: Enhance (Cu) X-ray Source	2610 reflections with *I* > 2σ(*I*)
graphite	*R*_int_ = 0.019
Detector resolution: 10.5081 pixels mm^-1^	θ_max_ = 77.6°, θ_min_ = 4.9°
ω scans	*h* = −29→31
Absorption correction: multi-scan (*CrysAlis PRO*; Oxford Diffraction, 2010)	*k* = −31→31
*T*_min_ = 0.805, *T*_max_ = 1.000	*l* = −6→11
8078 measured reflections	

### Refinement

**Table:**

Refinement on *F*^2^	Primary atom site location: structure-invariant direct methods
Least-squares matrix: full	Secondary atom site location: difference Fourier map
*R*[*F*^2^ > 2σ(*F*^2^)] = 0.043	Hydrogen site location: inferred from neighbouring sites
*wR*(*F*^2^) = 0.134	H atoms treated by a mixture of independent and constrained refinement
*S* = 1.08	*w* = 1/[σ^2^(*F*_o_^2^) + (0.0876*P*)^2^ + 0.4988*P*] where *P* = (*F*_o_^2^ + 2*F*_c_^2^)/3
3109 reflections	(Δ/σ)_max_ = 0.001
189 parameters	Δρ_max_ = 0.16 e Å^−^^3^
0 restraints	Δρ_min_ = −0.21 e Å^−^^3^

### Special details

**Table:**

Geometry. Bond distances, angles *etc*. have been calculated using the rounded fractional coordinates. All su's are estimated from the variances of the (full) variance-covariance matrix. The cell e.s.d.'s are taken into account in the estimation of distances, angles and torsion angles
Refinement. Refinement of *F*^2^ against ALL reflections. The weighted *R*-factor *wR* and goodness of fit *S* are based on *F*^2^, conventional *R*-factors *R* are based on *F*, with *F* set to zero for negative *F*^2^. The threshold expression of *F*^2^ > 2σ(*F*^2^) is used only for calculating *R*-factors(gt) *etc*. and is not relevant to the choice of reflections for refinement. *R*-factors based on *F*^2^ are statistically about twice as large as those based on *F*, and *R*- factors based on ALL data will be even larger.

### Fractional atomic coordinates and isotropic or equivalent isotropic displacement parameters (Å^2^)

**Table:**

	*x*	*y*	*z*	*U*_iso_*/*U*_eq_	Occ. (<1)
N1	0.00564 (4)	0.01347 (4)	0.24242 (9)	0.0428 (3)	
N3	0.09186 (4)	0.00845 (4)	0.23202 (10)	0.0452 (3)	
C2	0.04817 (4)	−0.01899 (5)	0.23981 (11)	0.0412 (3)	
C4	0.07726 (5)	0.06023 (5)	0.22951 (13)	0.0487 (3)	
C5	0.02420 (5)	0.06453 (5)	0.23682 (13)	0.0488 (3)	
C11	−0.04871 (4)	−0.00047 (5)	0.25460 (11)	0.0421 (3)	
C12	−0.07965 (5)	−0.00048 (6)	0.13375 (13)	0.0582 (5)	
C13	−0.13211 (5)	−0.01271 (7)	0.14490 (16)	0.0649 (5)	
C14	−0.15362 (5)	−0.02441 (7)	0.27589 (17)	0.0618 (4)	
C15	−0.12261 (5)	−0.02413 (7)	0.39614 (15)	0.0627 (5)	
C16	−0.06994 (5)	−0.01241 (6)	0.38579 (12)	0.0526 (4)	
C21	0.04616 (5)	−0.07654 (5)	0.24358 (12)	0.0473 (3)	
C22	0.01046 (6)	−0.10535 (6)	0.16451 (16)	0.0597 (4)	
C23	0.01189 (7)	−0.15977 (6)	0.1680 (2)	0.0778 (6)	
C24	0.04837 (8)	−0.18527 (7)	0.2490 (2)	0.0856 (7)	
C25	0.08410 (8)	−0.15716 (7)	0.3262 (2)	0.0869 (7)	
C26	0.08325 (6)	−0.10288 (6)	0.32454 (17)	0.0662 (5)	
C41	0.11729 (7)	0.10273 (6)	0.2167 (2)	0.0696 (5)	
C51	−0.01126 (7)	0.11082 (6)	0.2437 (2)	0.0704 (5)	
O1W	0.24177 (4)	0.04630 (4)	0.44390 (11)	0.0590 (3)	
H12	−0.06522	0.00770	0.04549	0.0699*	
H13	−0.15303	−0.01307	0.06375	0.0779*	
H14	−0.18902	−0.03247	0.28311	0.0742*	
H15	−0.13718	−0.03186	0.48453	0.0753*	
H16	−0.04894	−0.01257	0.46680	0.0631*	
H22	−0.01446	−0.08828	0.10917	0.0716*	
H23	−0.01212	−0.17894	0.11479	0.0933*	
H24	0.04891	−0.22165	0.25155	0.1027*	
H25	0.10914	−0.17460	0.38022	0.1043*	
H26	0.10756	−0.08410	0.37773	0.0794*	
H41A	0.15164	0.08752	0.21390	0.1045*	0.500
H41B	0.11467	0.12573	0.29733	0.1045*	0.500
H41C	0.11126	0.12219	0.13073	0.1045*	0.500
H41D	0.10007	0.13611	0.21408	0.1045*	0.500
H41E	0.13704	0.09790	0.13064	0.1045*	0.500
H41F	0.14045	0.10144	0.29724	0.1045*	0.500
H51A	−0.04695	0.09918	0.24795	0.1055*	0.500
H51B	−0.00634	0.13205	0.16032	0.1055*	0.500
H51C	−0.00332	0.13102	0.32724	0.1055*	0.500
H51D	0.00921	0.14232	0.24239	0.1055*	0.500
H51E	−0.03140	0.10945	0.33002	0.1055*	0.500
H51F	−0.03442	0.11048	0.16310	0.1055*	0.500
H1W	0.2423 (7)	0.0816 (7)	0.4596 (18)	0.064 (4)*	
H2W	0.2563 (9)	0.0340 (9)	0.520 (2)	0.095 (7)*	

### Atomic displacement parameters (Å^2^)

**Table:**

	*U*^11^	*U*^22^	*U*^33^	*U*^12^	*U*^13^	*U*^23^
N1	0.0387 (5)	0.0480 (5)	0.0416 (5)	−0.0005 (4)	−0.0003 (3)	0.0024 (4)
N3	0.0396 (5)	0.0533 (5)	0.0427 (4)	−0.0041 (4)	0.0026 (4)	0.0011 (4)
C2	0.0382 (5)	0.0498 (6)	0.0357 (5)	−0.0006 (4)	0.0019 (4)	0.0033 (4)
C4	0.0484 (6)	0.0510 (6)	0.0468 (6)	−0.0068 (5)	0.0022 (5)	−0.0010 (5)
C5	0.0512 (6)	0.0466 (6)	0.0485 (6)	−0.0023 (5)	−0.0005 (5)	0.0010 (5)
C11	0.0363 (5)	0.0498 (6)	0.0403 (5)	0.0019 (4)	−0.0005 (4)	0.0015 (4)
C12	0.0493 (7)	0.0851 (10)	0.0402 (6)	−0.0004 (6)	−0.0044 (5)	0.0102 (6)
C13	0.0463 (7)	0.0911 (10)	0.0574 (7)	0.0015 (6)	−0.0156 (6)	0.0082 (7)
C14	0.0370 (6)	0.0771 (9)	0.0713 (8)	−0.0010 (5)	−0.0022 (6)	0.0031 (7)
C15	0.0496 (7)	0.0881 (10)	0.0505 (7)	−0.0062 (6)	0.0081 (5)	0.0060 (7)
C16	0.0457 (6)	0.0745 (8)	0.0377 (5)	−0.0039 (5)	−0.0026 (4)	0.0032 (5)
C21	0.0448 (6)	0.0486 (6)	0.0486 (6)	0.0008 (4)	0.0111 (5)	0.0060 (5)
C22	0.0538 (7)	0.0548 (7)	0.0705 (8)	−0.0052 (5)	0.0082 (6)	−0.0030 (6)
C23	0.0768 (10)	0.0573 (8)	0.0992 (12)	−0.0150 (7)	0.0224 (9)	−0.0099 (8)
C24	0.0900 (12)	0.0491 (8)	0.1177 (15)	0.0020 (8)	0.0344 (11)	0.0162 (9)
C25	0.0846 (12)	0.0666 (10)	0.1096 (14)	0.0172 (9)	0.0127 (11)	0.0349 (10)
C26	0.0621 (8)	0.0636 (8)	0.0729 (9)	0.0066 (6)	0.0011 (7)	0.0178 (7)
C41	0.0635 (8)	0.0572 (8)	0.0882 (10)	−0.0169 (6)	0.0119 (8)	−0.0054 (7)
C51	0.0643 (9)	0.0542 (8)	0.0926 (11)	0.0080 (6)	−0.0007 (8)	−0.0011 (7)
O1W	0.0640 (5)	0.0518 (6)	0.0611 (5)	−0.0050 (4)	−0.0054 (4)	−0.0043 (4)

### Geometric parameters (Å, °)

**Table:**

O1W—H2W	0.86 (2)	C12—H12	0.9300
O1W—H1W	0.914 (18)	C13—H13	0.9300
N1—C5	1.3891 (16)	C14—H14	0.9300
N1—C2	1.3672 (15)	C15—H15	0.9300
N1—C11	1.4381 (15)	C16—H16	0.9300
N3—C4	1.3748 (16)	C22—H22	0.9300
N3—C2	1.3202 (15)	C23—H23	0.9300
C2—C21	1.4717 (18)	C24—H24	0.9300
C4—C41	1.497 (2)	C25—H25	0.9300
C4—C5	1.3618 (18)	C26—H26	0.9300
C5—C51	1.491 (2)	C41—H41B	0.9600
C11—C12	1.3819 (16)	C41—H41A	0.9600
C11—C16	1.3789 (16)	C41—H41E	0.9600
C12—C13	1.3803 (18)	C41—H41C	0.9600
C13—C14	1.379 (2)	C41—H41D	0.9600
C14—C15	1.378 (2)	C41—H41F	0.9600
C15—C16	1.3820 (18)	C51—H51B	0.9600
C21—C26	1.388 (2)	C51—H51C	0.9600
C21—C22	1.387 (2)	C51—H51E	0.9600
C22—C23	1.391 (2)	C51—H51F	0.9600
C23—C24	1.368 (3)	C51—H51D	0.9600
C24—C25	1.369 (3)	C51—H51A	0.9600
C25—C26	1.387 (2)		
			
O1W···O1W^i^	2.8957 (15)	C41···H51D	2.9500
O1W···O1W^ii^	2.8957 (15)	C51···H41D	2.9300
O1W···N3^ii^	2.9111 (14)	H1W···N3^ii^	2.010 (18)
O1W···H14^iii^	2.8200	H1W···H41A^ii^	2.4700
O1W···H2W^i^	2.03 (2)	H1W···C4^ii^	2.910 (18)
O1W···H13^iv^	2.7900	H1W···C2^ii^	3.098 (18)
O1W···H15^v^	2.7800	H2W···O1W^ii^	2.03 (2)
O1W···H41A^ii^	2.7900	H2W···H14^v^	2.5200
O1W···H14^v^	2.9100	H12···N3^vi^	2.7200
N3···O1W^i^	2.9111 (14)	H12···C2^vi^	2.7300
N1···H16^v^	2.9400	H13···O1W^viii^	2.7900
N1···H22	2.9300	H14···O1W^vii^	2.8200
N3···H26	2.7600	H14···O1W^v^	2.9100
N3···H1W^i^	2.010 (18)	H14···H2W^v^	2.5200
N3···H12^vi^	2.7200	H15···O1W^v^	2.7800
C11···C22	3.191 (2)	H16···C2^v^	2.8700
C12···C22	3.544 (2)	H16···N1^v^	2.9400
C12···C51	3.493 (2)	H22···N1	2.9300
C22···C12	3.544 (2)	H22···C11	2.7700
C22···C11	3.191 (2)	H22···C12	2.8000
C23···C25^vii^	3.597 (3)	H24···C24^iii^	3.0800
C25···C23^iii^	3.597 (3)	H24···H51C^i^	2.5600
C51···C12	3.493 (2)	H25···C23^iii^	2.9600
C2···H1W^i^	3.098 (18)	H25···C22^iii^	3.0600
C2···H16^v^	2.8700	H26···N3	2.7600
C2···H12^vi^	2.7300	H41A···H1W^i^	2.4700
C4···H1W^i^	2.910 (18)	H41A···O1W^i^	2.7900
C11···H22	2.7700	H41B···C24^ii^	2.9900
C11···H51A	2.5500	H41B···C25^ii^	2.8600
C11···H51E	2.9300	H41B···C26^ii^	2.9200
C11···H51F	2.9800	H41D···C51	2.9300
C12···H51F	3.0700	H41D···H51D	2.3400
C12···H22	2.8000	H41F···C26^ii^	3.0900
C12···H51A	2.8900	H51A···C11	2.5500
C22···H25^vii^	3.0600	H51A···C12	2.8900
C23···H25^vii^	2.9600	H51C···C24^ii^	2.9800
C24···H51C^i^	2.9800	H51C···H24^ii^	2.5600
C24···H41B^i^	2.9900	H51D···C41	2.9500
C24···H24^vii^	3.0800	H51D···H41D	2.3400
C25···H41B^i^	2.8600	H51E···C11	2.9300
C26···H41F^i^	3.0900	H51F···C11	2.9800
C26···H41B^i^	2.9200	H51F···C12	3.0700
			
H1W—O1W—H2W	102.7 (18)	C26—C25—H25	120.00
C2—N1—C11	128.22 (10)	C24—C25—H25	120.00
C5—N1—C11	124.42 (10)	C25—C26—H26	120.00
C2—N1—C5	107.32 (10)	C21—C26—H26	120.00
C2—N3—C4	106.41 (10)	C4—C41—H41A	109.00
N1—C2—N3	110.54 (11)	C4—C41—H41C	109.00
N1—C2—C21	125.30 (10)	C4—C41—H41D	109.00
N3—C2—C21	124.15 (10)	C4—C41—H41B	109.00
N3—C4—C41	120.94 (12)	C4—C41—H41F	109.00
C5—C4—C41	128.74 (12)	H41A—C41—H41B	109.00
N3—C4—C5	110.30 (11)	H41A—C41—H41C	109.00
N1—C5—C51	122.40 (12)	H41A—C41—H41D	141.00
C4—C5—C51	132.14 (13)	H41A—C41—H41E	56.00
N1—C5—C4	105.43 (11)	H41A—C41—H41F	56.00
N1—C11—C16	120.37 (10)	C4—C41—H41E	109.00
C12—C11—C16	120.46 (11)	H41B—C41—H41D	56.00
N1—C11—C12	119.16 (10)	H41B—C41—H41E	141.00
C11—C12—C13	119.56 (12)	H41B—C41—H41F	56.00
C12—C13—C14	120.25 (13)	H41C—C41—H41D	56.00
C13—C14—C15	119.93 (12)	H41C—C41—H41E	56.00
C14—C15—C16	120.22 (13)	H41C—C41—H41F	141.00
C11—C16—C15	119.58 (11)	H41D—C41—H41E	109.00
C2—C21—C26	118.27 (11)	H41D—C41—H41F	109.00
C22—C21—C26	118.95 (13)	H41E—C41—H41F	109.00
C2—C21—C22	122.71 (11)	H41B—C41—H41C	109.00
C21—C22—C23	120.04 (14)	C5—C51—H51B	109.00
C22—C23—C24	120.47 (16)	C5—C51—H51C	109.00
C23—C24—C25	119.90 (17)	C5—C51—H51A	109.00
C24—C25—C26	120.55 (17)	C5—C51—H51E	109.00
C21—C26—C25	120.10 (15)	C5—C51—H51F	109.00
C13—C12—H12	120.00	C5—C51—H51D	109.00
C11—C12—H12	120.00	H51A—C51—H51C	109.00
C14—C13—H13	120.00	H51A—C51—H51D	141.00
C12—C13—H13	120.00	H51A—C51—H51E	56.00
C13—C14—H14	120.00	H51A—C51—H51F	56.00
C15—C14—H14	120.00	H51B—C51—H51C	109.00
C16—C15—H15	120.00	H51B—C51—H51D	56.00
C14—C15—H15	120.00	H51B—C51—H51E	141.00
C11—C16—H16	120.00	H51B—C51—H51F	56.00
C15—C16—H16	120.00	H51C—C51—H51D	56.00
C23—C22—H22	120.00	H51C—C51—H51E	56.00
C21—C22—H22	120.00	H51C—C51—H51F	141.00
C22—C23—H23	120.00	H51D—C51—H51E	109.00
C24—C23—H23	120.00	H51D—C51—H51F	109.00
C23—C24—H24	120.00	H51E—C51—H51F	109.00
C25—C24—H24	120.00	H51A—C51—H51B	109.00
			
C5—N1—C2—N3	−0.41 (12)	N3—C4—C5—N1	−0.66 (13)
C5—N1—C2—C21	−179.82 (10)	N3—C4—C5—C51	177.15 (14)
C11—N1—C2—N3	−178.01 (9)	C41—C4—C5—N1	177.86 (13)
C11—N1—C2—C21	2.58 (17)	C41—C4—C5—C51	−4.3 (2)
C2—N1—C5—C4	0.65 (12)	N1—C11—C12—C13	−178.61 (13)
C2—N1—C5—C51	−177.43 (12)	C16—C11—C12—C13	−0.2 (2)
C11—N1—C5—C4	178.36 (10)	N1—C11—C16—C15	177.99 (13)
C11—N1—C5—C51	0.28 (18)	C12—C11—C16—C15	−0.4 (2)
C2—N1—C11—C12	−101.64 (14)	C11—C12—C13—C14	0.6 (2)
C2—N1—C11—C16	79.95 (16)	C12—C13—C14—C15	−0.3 (3)
C5—N1—C11—C12	81.14 (15)	C13—C14—C15—C16	−0.3 (3)
C5—N1—C11—C16	−97.27 (15)	C14—C15—C16—C11	0.7 (2)
C4—N3—C2—N1	0.00 (13)	C2—C21—C22—C23	177.34 (13)
C4—N3—C2—C21	179.42 (10)	C26—C21—C22—C23	0.5 (2)
C2—N3—C4—C5	0.42 (13)	C2—C21—C26—C25	−177.29 (14)
C2—N3—C4—C41	−178.24 (12)	C22—C21—C26—C25	−0.3 (2)
N1—C2—C21—C22	42.85 (17)	C21—C22—C23—C24	0.0 (3)
N1—C2—C21—C26	−140.30 (12)	C22—C23—C24—C25	−0.6 (3)
N3—C2—C21—C22	−136.48 (13)	C23—C24—C25—C26	0.8 (3)
N3—C2—C21—C26	40.36 (17)	C24—C25—C26—C21	−0.4 (3)

Symmetry codes: (i) −*y*+1/4, *x*−1/4, *z*−1/4; (ii) *y*+1/4, −*x*+1/4, *z*+1/4; (iii) *y*+1/4, −*x*−1/4, −*z*+3/4; (iv) −*y*+1/4, *x*+1/4, −*z*+1/4; (v) −*x*, −*y*, −*z*+1; (vi) −*x*, −*y*, −*z*; (vii) −*y*−1/4, *x*−1/4, −*z*+3/4; (viii) *y*−1/4, −*x*+1/4, −*z*+1/4.

### Hydrogen-bond geometry (Å, °)

**Table:**

*D*—H···*A*	*D*—H	H···*A*	*D*···*A*	*D*—H···*A*
O1W—H1W···N3^ii^	0.914 (18)	2.010 (18)	2.9111 (14)	168.6 (16)
O1W—H2W···O1W^ii^	0.86 (2)	2.03 (2)	2.8957 (15)	175 (2)

Symmetry codes: (ii) *y*+1/4, −*x*+1/4, *z*+1/4.
